# Decadal predictions of future habitat favourability of the European green crab (*Carcinus maenas*) on the Pacific Coast of North America

**DOI:** 10.1007/s10750-026-06149-4

**Published:** 2026-04-23

**Authors:** D. A. Villar, Aaron Clark-Ginsberg, Rebecca Tisherman

**Affiliations:** 1 Department of Anthropology, Durham University, Durham, County Durham UK; 2 Department of Biology, University of Oxford, Oxford, Oxfordshire UK; 3 RAND Corporation, Santa Monica, CA USA; 4 RAND Corporation, Pittsburgh, PA USA

**Keywords:** Climate change, Marine invasive species, Bayesian distribution modelling, Fisheries, Invertebrate invasive

## Abstract

The European green crab (*Carcinus maenas*) is a globally important invasive species. Since 1989, it has been spreading north from San Francisco Bay on the Pacific Coast of North America. Climate change is likely to lead to the species spreading further north into Alaskan and Arctic waters. Previous studies predicting its potential future distribution have focused on its range at the end of the century, under only one of multiple climate change scenarios. We used Bayesian Additive Regression Trees (BART) and benthic environmental variable predictors derived from the latest Intergovernmental Panel on Climate Change report to produce decadal predictions under four climate change scenarios; SSP1, SSP2, SSP4, and SSP5. We find that under all but the lowest emission scenarios, the European green crab will find suitable habitat as far north as the Seward Peninsula, and potentially, under the highest emission scenarios, into the Arctic Circle. This could have devastating consequences for the local ecology of the Pacific Coast of North America, for local economies dependent on fisheries, and for local communities, particularly indigenous communities. Increased monitoring in regions where green crab is likely to find favourable habitat could help with early detection, and potentially early functional eradication before populations establish themselves.

**Supplementary Information:**

The online version contains supplementary material available at 10.1007/s10750-026-06149-4.

## Introduction

Aquatic Invasive Species (AIS) are one of the major threats to marine biodiversity globally (Havel et al., 2015; Gallardo et al., 2016). They are associated with the loss of billions of USD a year to the global economy (Cuthbert et al., 2021), due to their direct impact on commercially valuable marine species, and indirect habitat degradation. The European green crab is amongst the most pervasive AIS on the planet, with populations established on every continent except Antarctica (Ens et al., 2022; Frederich & Lancaster, 2024). It was first observed on the Pacific Coast of North America in the 1980s (Cohen et al., 1995), established itself in British Columbia by 1997 (Gillespie et al., 2015), and had established in southeastern parts of Alaska as early as 2014 (Davis, 2022), threatening native species and potentially continuing to move north further into Alaskan waters. Due to anthropogenic climate change, the waters in northerly latitudes are warming faster than the global average (Donney et al., 2012), and increasing more rapidly than what was originally predicted in climate change models (Meier et al., 2014). The purpose of this study is to provide decadal predictions of the distribution of the European green crab on the Pacific Coast of North America from 2030 to 2100.

Most AIS in marine environments are transported by humans accidently through cargo ships, especially with ballast (Bailey, 2015). Eradication of invasive species of any kind, but particularly of marine ones, is practically impossible once a population is established (Locke & Hanson, 2009). This means that invasions have to be prevented before they begin, either by preventing individuals of the invasive species from initially entering the region, or rapidly extirpating individuals when they arrive to the point of functional eradication. Such proactive management strategies are expensive, and often difficult to fund in fields like conservation biology where money is the primary limiting resource (Bottrill et al., 2008). One way to prioritise monitoring resources is through knowing which regions are more vulnerable to invasion of a particular species, and, under changing climatic conditions, when those regions are expected to become more vulnerable (Elith, 2017). Species Distribution Models (SDMs) are a powerful tool which predict the habitat favourability of a region for a species based on abiotic factors (Elith & Leathwick, 2009). SDM’s assess the vulnerability of regions to invasion either under present conditions (Pěknicová & Berchová-Bímová, 2016) or under future climatic conditions (de Rivera et al., 2011; Luizza et al., 2016; Goldsmit et al., 2018, 2020).

The European green crab *Carcinus maenas* (L., 1758) is native to Europe and North Africa (Frederich & Lancaster, 2024), where it can be split into two fairly distinct populations: a northern population and a southern one (Compton et al., 2010; Frederich & Lancaster, 2024). It was first reported outside its native range on the Atlantic Coast of North America in 1817 (Say, 1817), and by 1900 had been reported as far afield as Australia, Sri Lanka, and Panama, though only in North America and Australia (Walton et al. 2002) did it manage to establish a self-sustaining invasive population (Carlton & Cohen, 2003). Its invasiveness has been attributed to its ability to survive for long periods without food, either in ship’s ballast or bored into wood (Carlton & Cohen, 2003; Klassen & Locke, 2007), and to its rapid life history, especially in the most motile larval stage (deRivera et al., 2007; Brasseale et al., 2019; Yamada et al., 2022), which enables it to quickly colonise suitable habitat. Globally, the main drivers of its invasive range are water temperature and suitable currents (Compton et al., 2010; Yamada & Kosro, 2010; Yamada et al., 2021, 2022). The lack of favourable currents has confined it to a relatively small part of its potential range in South Africa (Ens et al., 2022; Frederich & Lancaster, 2024) and might be limiting its expansion in South America (Malvé et al., 2025). In addition to these abiotic drivers of its range expansion, the European green crab’s ability to colonise a region can depend on whether the source population comes from its native southern or northern native range, with those from the northern part of its native range showing a greater cold tolerance (Compton et al., 2010; Frederich & Lancaster, 2024).

European green crab was first observed on the Pacific Coast of North America in San Francisco Bay, in 1989 (Cohen et al., 1995). Initially some authors assumed that the species would not be able to establish a permanent population (Compton et al., 2010, but see Yamada & Gillespie, 2008 for an opposing contemporary opinion). Yet, a warmer than average El Niño in 1997–1998 permitted a rapid expansion of the European green crab from San Francisco up the Oregon and Washington Coasts, to the west coast of Vancouver Island (deRivera et al., 2007; Gillespie et al., 2015). Over the next two decades after the 1997–1998 El Niño year, the species established itself further up the coast of British Columbia, with most rapid year-to-year northwards movement being associated with warmer years (Gillespie et al., 2015). The European green crab was primarily concentrated in sheltered islets on these more northern parts of its invasive range until another warm El Niño in 2014 allowed it to cross the Strait of Juan de Fuca and spread into the Salish Sea (Ens et al., 2022). Since then, the European green crab has established itself in the Salish Sea, and has moved further north, to the northern coast of British Columbia (Brasseale et al., 2019; Yamada et al., 2021; Ens et al., 2022; Engel et al., 2025), to Haida Gwaii (Yamada et al., 2021), and most recently to the southeastern parts of Alaska (Davis, 2022).

On Pacific Coast of North America, European green crabs can have significant negative impacts on local ecosystems and on the economies which rely on them. European green crabs are considered a threat to commercially important decapods and shellfish (McDonald et al., 2001), and can reduce eelgrass *Zostera* sp. (L. 1758) cover by between 50 and 100%, due to their burrowing and direct consumption (Howard et al., 2019; Ens et al., 2022). Eelgrass is important spawning habitat for commercially important fish such as Pacific Herring *Clupea pallasii* (Valenciennes 1847) and provides foraging grounds for fish such as the Chinook Salmon *Oncorhynchus tshawytscha* (Walbaum 1792), (Shelton et al., 2014; Kennedy et al., 2018; Howard et al., 2019) which is both commercially important (Wilson, 2025), and culturally significant to the indigenous communities of the Pacific Coast of North America in Alaska, British Columbia, Washington, Oregon, and California (Holen, 2014; Carothers et al., 2021; Connoy et al., 2024). Eelgrass is also important for reducing coastal erosion and thus protects both littoral habitats and coastal communities (Hamminga & Duarte, 2000). The economic cost of the European green crab on the northeastern Pacific coast can be difficult to measure directly, and there have not been systematic attempts to study the economic cost of the European green crab on all fisheries in the our entire study region. However estimates from Canada and the United States put the economic cost of the European green crab on fisheries in a range from 0.9 million USD to 114.29 million USD (Colautti et al. 2006; Grosholz et al., 2011; Ens et al., 2022), depending on the region, fishery, and pricing mechanism used. However, all estimates which have looked at potential costs should the European green crab’s range expand agree that economic cost is expected to worsen as the European green crab moves north to Alaska, which has more economically valuable softshell shellfish fisheries than those in British Columbia and the southern US Pacific Coast which it has already invaded.

As early as the 1950s, some authors attributed the northwards shift of the European green crab from the New York area in 1817 to the Atlantic Provinces of Canada by 1900 to increasing ocean temperatures (Carson, 1955; Welch, 1968). However, that hypothesis remains unproven, and it is possible that this northwards expansion was more due to post invasion infilling than the early stages of ocean temperature increases following the end of the Little Ice Age (Gebbie, 2019). Early predictions about polewards shifts of the European green crab were sanguine, both because the population on the Pacific Coast is primarily derived from Southern Europe (Compton et al., 2010), and because of the multiple barriers between San Francisco Bay and the more northern coasts of North America. Unfortunately, these predictions have not proven true, as can be seen by its expansion, mediated in part by warmer El Niños than in recent decades compared to the twentieth century norm (de Rivera et al., 2007; Yamada & Gillespie, 2008; Yamada & Kosro, 2010; Brasseale et al., 2019; Yamada et al., 2022). Climate change may facilitate faster spread of the European green crab, as high-latitude marine waters are warming more than the global average (Doney et al., 2012).

SDMs predicting potential future range of the European green crab on the Pacific Coast of North America showed that there was potentially suitable habitat for the European green crab as far north as 60–65° N, both in the present when the studies took place and by the end of the twenty-first century under median climate change scenarios (Compton et al., 2010; De Rivera et al., 2011; Goldsmit et al., 2020). A study focused on the physiological temperature tolerance of the European green crab suggested a northernmost range of 60.5° N, though it did suggest that under climate change Bristol Bay and the Aleutian Islands could also become suitable habitat for the species (Kelley et al., 2013). However, these studies only focused on the end of century predictions of the range of the European green crab, not looking at potential decadal change in its distribution. They also used climate predictions from the IPCC Fourth Assessment Report or IPCC Fifth Assessment Report (Intergovernmental Panel on Climate Change, 2007; Intergovernmental Panel on Climate Change, 2015), rather than the newest IPCC Sixth Assessment Report (Intergovernmental Panel on Climate Change, 2023), which includes more possible climate change scenarios. The SDM studies also converted probabilistic SDM predictions to binary maps through arbitrary thresholds, such as minimum training presence (Phillips et al., 2006). This practice, while intuitive and common, increases the arbitrariness of predictions and removes significant amounts of information which the probabilistic raw model output provides.

This study uses Bayesian SDMs and the latest environmental data to characterize the future distribution of the European green crab on the Pacific Coast of North America. The results of this study will provide fisheries managers a finer-scale temporal resolution for predicting when European green crabs can be expected to become a problem in their region, and allow them to focus their monitoring efforts accordingly in both time and space.

## Methods

### Study region

**Fig. 1:**
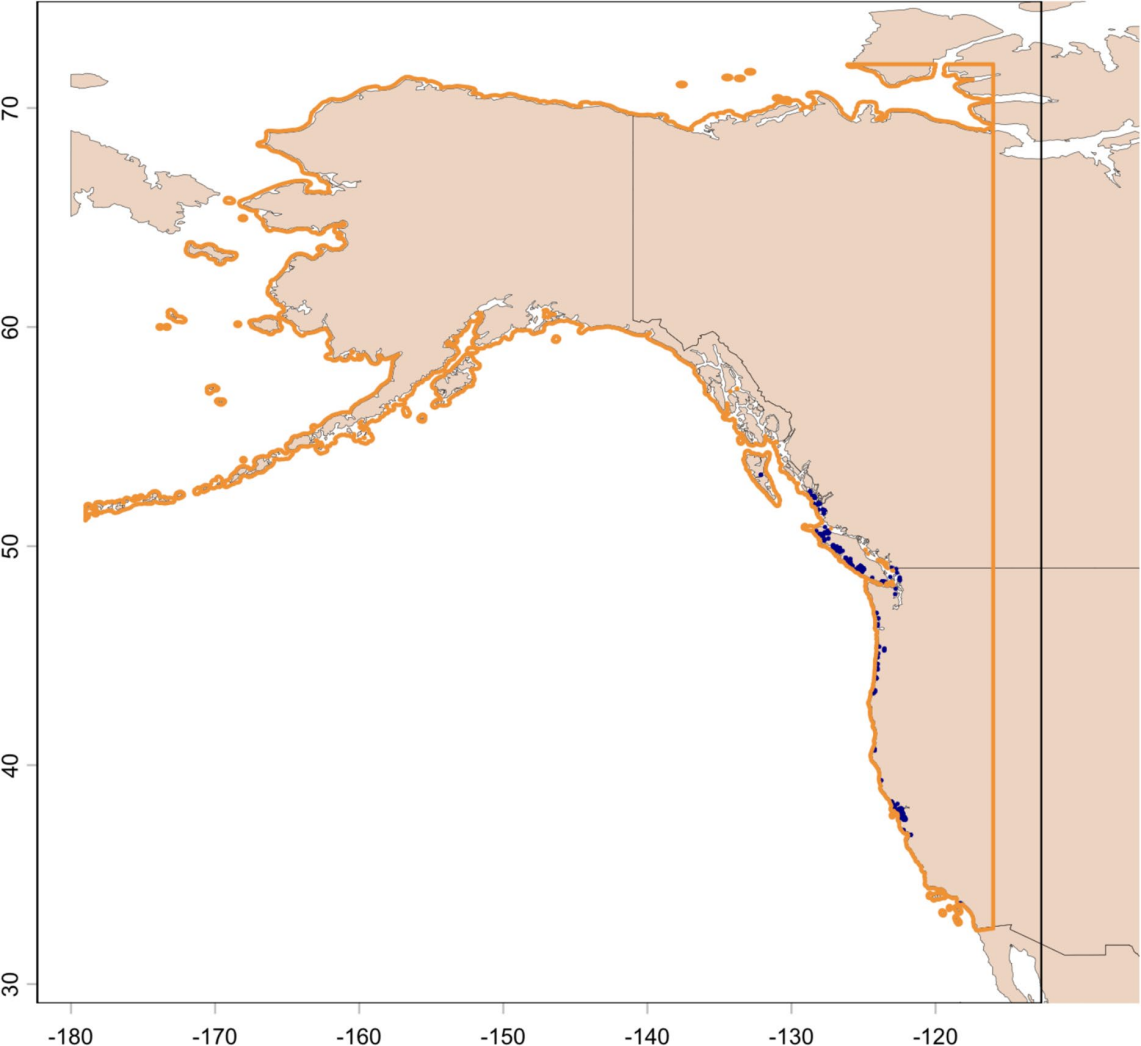
Study region. 10 km buffer from the Pacific Coast of North America from California to Alaska is outlined in orange. European Green crab Presence Points between 2010 and 2020 are blue dots on the map

While SDMs are a powerful tool for predicting present and future habitat suitability, their predictive power is often geographically restricted. When the study region is too small, the model can be overfitted to the study region and not accurately predict habitat suitability across other parts of the species’ range (Elith et al., 2010). However, in cases where the study region is too large, SDMs can overpredict the distribution of species, as it ignores non-environmental barriers to species presence, such as geographic barriers which prevent dispersal (Velazco et al., 2020). This can especially be a problem when modelling habitat favourability in invasive species, as models based on the native range can often not be accurate for assessing the invasive range, or vice versa (Nguyen & Leung, 2022). Common approaches to solving these problems include limiting SDM model creation and predictions to a region within the buffered minimum convex hull polygon of known presence points of a species (Meyer et al., 2017) or combining information about distribution capacity of species with information about their dispersal patterns and abilities (Merow et al., 2011). However, these techniques utility depends either on detailed knowledge on the movement ecology of the species or on the entire region of interest being within the convex hull of presence points, neither of which is true for European green crab on the Pacific Coast. In this study, we are primarily concerned with changing habitat favourability in order to give wildlife and fisheries managers a warning about when green crab could become a problem. We are therefore less concerned with the problems associated with model overpredicting than we are with problems associated with model overfitting. We have subsequently selected a 10 km buffer region from the North American coast, from the Mexico-USA border to Alaska, as our study extent for both model building and model prediction (Fig. 1). This gave us a total of 26,335 raster cells covering an area of 415,217.5 km^2^. To test the effect of the buffer size on our model, we also tested our model using 50, 25, and 100 km buffers from the coast. The results of these analyses can be found in the supplemental materials. All analysis in this paper was done in R version 4.4.3 (R Core Team, 2025).

### Species presences and environmental covariates

Species Presences were obtained from the Global Biodiversity Information Facility (2020). Presence points from between 2010 and 2020, inclusive, were downloaded using the *sp_occurrence()* function from the geodata R library (Hijmans, 2021). These presences were then cleaned by removing absences, removing presences which duplicated the location of other presences, and removing presences whose coordinate uncertainty is greater than the resolution of our environmental rasters. We additionally obtained occurrence points from iNaturalist (2025), Ocean Biodiversity Information System (OBIS) (2025) using the *robis* R package (Provoost & Busch, 2017), and primary literature which had not been included in the aforementioned databases, collected on behalf of relevant authorities of coastal jurisdictions (Yamada & Kosro, 2010; Yamada & Randall, 2011; Yamada et al., 2013a, b, 2015, 2017, 2018, 2019, 2020; Yamada & Kaufman, 2014; Yamada & Randall, 2016; Fisheries and Oceans Canada, 2025). This gave us a total of 1041 presences; however, this was reduced to 52 presences in our model. This is because we could only take one presence per cell in our environmental raster. This is more than the 30 presence points commonly accepted as the minimum number of presence points required to create a robust SDM (Wisz et al., 2008). In addition to the presence points, we sampled 9948 pseudo-absences, for a total of 10,000 points used in our model (Barbet‐Massin et al., 2012). The pseudo-absences were sampled quasi-randomly, using a bias layer mapping human impact on the ocean for 2010 (Frazier, 2019), to try to control for any biases in survey effort or detection probability of the European green crab in the study region. We tested the impact of the use of the bias layer on our model by running each model selecting for pseudo-absences without the bias layer, and by varying the number of pseudo-absences, so that the total number of points (presences and pseudo-absences) were, respectively, 10,000, 5000, 1000, and 100. The results of each of these models were compared to each other using Schoener’s *D* (Schoener, 1970). A Schoener’s *D* of over 0.7 indicates significant similarity in model results (Schoener, 1970). This work made use of the Hamilton HPC Service of Durham University.

Environmental variables were obtained from Bio-ORACLE v3.0 (Assis et al., 2024). This is the latest edition of a standard global set of oceanic environmental variables. As the European green crab is a benthic species, we first downloaded benthic variables at the average depth for each pixel. The initial list of variables were: topographic slope, topographic aspect, topographic position index, terrain ruggedness index, and the mean, maximum, and minimum values of ocean temperature, salinity, sea water velocity, sea water direction, nitrate, phosphate, silicate, dissolved molecular oxygen, iron, primary productivity, pH, and water depth. These variables were then filtered by testing for multicollinearity, removing variables which had a variance inflation factor greater than 10 (Hair et al., 2019) and which had a Pearson correlation coefficient greater than 0.8 (Pearson, 1895). Remaining variables were removed using the *variable.step()* function from the embarcadero R library (Carlson, 2020). This function removes the least informative variable based on the root mean square error (RMSE) following 200 model iterations across 20 trees. It then provides which combination of potential explanatory variables can provide the most explanatory value with the lowest number of explanatory variables. All environmental variables are at a resolution of 0.05°, or an average of 15.5 km by 15.5 km within our study region.

### Habitat suitability modelling

We used Bayesian Additive Regression Trees (BART) (Chipman et al., 2010) to model the current and future habitat favourability of the study region for the European green crab. BART is a relatively newer SDM method compared to more established methods, such as MaxEnt (Phillips et al., 2006), Occupancy Modelling (MacKenzie et al., 2018), and Generalised Additive Models (GAM) (Naimi & Araújo, 2016). However, it is a very powerful method, originally used in ecology to study the distribution of zoonotic diseases (Carlson, 2020) but now seeing increasing use across various branches of ecology (Carlson et al., 2022; Baquero et al., 2021; Hanz et al., 2023; Lazagabaster et al., 2024). BART models consistently perform well when compared to more traditional SDM methods, such as GAM and MaxEnt (Carlson et al., 2022; Baquero et al., 2021; Dansereau et al., 2022), including ensembles of multiple SDMs (Lazagabaster et al., 2024). BART works by first fitting shallow “learner” trees, whose depth is controlled by three posterior distributions, namely the probability of the tree stopping at a node of a given depth, the probability of splitting a variable at a particular value, and the probability of a variable being drawn for a splitting rule. Once initial trees are fit, these are altered randomly by a Markov chain Monte Carlo (Robert & Casella, 2004) process. Predictions are done by sum-of-trees, as opposed to the Random Forest (Carlson, 2020) average of trees approach. Models were run using the default parameters of the embarcadero R package, namely 200 trees, 1000 posterior draws with a burn-in of 100 draws, and the default hyperparameters, namely power = 2.0, base = 0.95. In addition to its good performance and robustness, a benefit of using BART models is that it allows us to map the uncertainty in predictions. For each cell in the rasters we predicted, we extracted the 5th, 50th, and 95th percentile likelihood of the presence of the green crab according to our model.

Models were validated by spatial, or block, cross validation. Splitting the data spatially for cross validation in blocks is more robust than splitting it randomly, as the latter often underestimates prediction error compared to the former (Araújo et al., 2005). To do this spatial cross validation, the data was split into 5 k-folds. Each grid cell which was randomly assigned to one of these folds was 100 km × 100 km. For validation, a model was created with 20% of the dataset and then used to predict across the remaining 80%. We did this rather than the usual approach of using a model trained on a random 80% of the data to predict the remaining 20% since it gives a more conservative estimate of model performance. To evaluate model performance, we used three metrics: Area Under the receiver operating characteristic Curve (AUC) (Swets, 1988), True Skill Statistic (TSS) (Allouche et al., 2006), and Miller’s calibration slope (Miller et al., 1991). AUC is a measure of overall discrimination performance, TSS of classification performance, and Miller’s calibration slope of model reliability. All three are commonly used to assess SDMs (e.g., Baquero et al., 2021; Villar et al., 2024). The values of the AUC, TSS, and Miller’s calibration slope were then averaged across the spatial cross-validation folds to provide the metrics for the model. The averaged AUC, TSS, and Miller’s Calibration curve values were those for the test portion of the data, excluding the AUC, TSS, and Miller’s Calibration curve values of the training set (Fielding & Bell, 1997).

### Predicting future habitat suitability and change over time

While climate change is already significantly altering the distribution of species across the planet, the extent of future impacts of climate change remains uncertain and depends on the actions which countries take in the coming decades. The IPCC has labelled the various potential future paths of climate change as Shared Socioeconomic Pathways (SSPs) (Intergovernmental Panel on Climate Change, 2023). These range from SSP1, with the lowest future greenhouse emissions, to SSP5, with the highest. In this study, we looked at four SSPs: SSP1, SSP2, SSP4, and SSP5. SSP1, which assumes all countries meet their statutory obligations to reduce greenhouse gas emissions under the Paris Climate Accords, would see total warming stay under 2 °C by 2100. SSP2 is a “business as usual” scenario, where greenhouse gas emissions decline at the same speed as they have been doing for the first quarter of the century, and would see total warming of 4 °C by 2100. SSP4 is one where there is increasing inequality in greenhouse gas emissions between countries which are early adopters of renewable energy and other countries which double down on the use of fossil fuels. This scenario sees warming of 6 °C by 2100. SSP5 is the worst-case scenario, where the entire globe undergoes intensification of its use of fossil fuels, leading to an 8.5 °C temperature increase by 2100. We fitted our BART model to rasters for each decade between 2030 and 2100.

Rather than using an arbitrary threshold (Liu et al., 2005) to convert the continuous presence probability rasters, as is the norm in many studies which use SDMs to predict future habitat favourability for species under climate change (e.g., Delean et al., 2013; Lavender et al., 2021), we used fuzzy logic and fuzzy set theory (Zadeh, 1965) to look at how habitat suitability changed over time under different climate scenarios for the European green crab. Fuzzy logic is increasingly used in biogeography and spatial ecology, both to remove a level of arbitrariness which can significantly alter results and model interpretations which can result from thresholding (Hellegers et al., 2025), and to preserve the totality of the information which continuous habitat suitability rasters provide (Baquero et al., 2021; Li et al., 2024). This was done using the *fuzzyRangeChange()* function from the fuzzySim R package (Barbosa, 2018). We measured change in habitat suitability decadally as the proportional balance of raster cells which became more suitable minus those which became less suitable.

In addition to the R libraries which have already been mentioned, the R packages terra, predicts, collinear, modEvA, blockCV, and ggplot2 (Barbosa et al., 2016; Wickham, 2016; Valavi et al., 2019; Hijmans, 2020; Benito, 2023) and the ArcGIS Pro software were also used either for data analysis or for data visualisation.

## Results

**Fig. 2:**
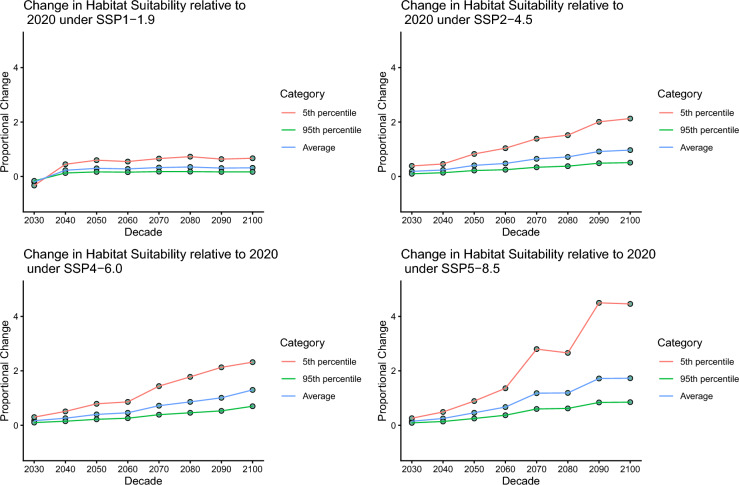
Decadal change in habitat favourability of the Pacific Coast of North America for the European Green crab, including uncertainty, relative to the 2010–2020 base. Average refers to the 50th percentile of change in habitat favourability relative to this baseline. Proportional change is based on the net percentage of grid cells whose change in habitat suitability was beyond the margin of error of habitat suitability in the 2010–2020 baseline

**Fig. 3:**
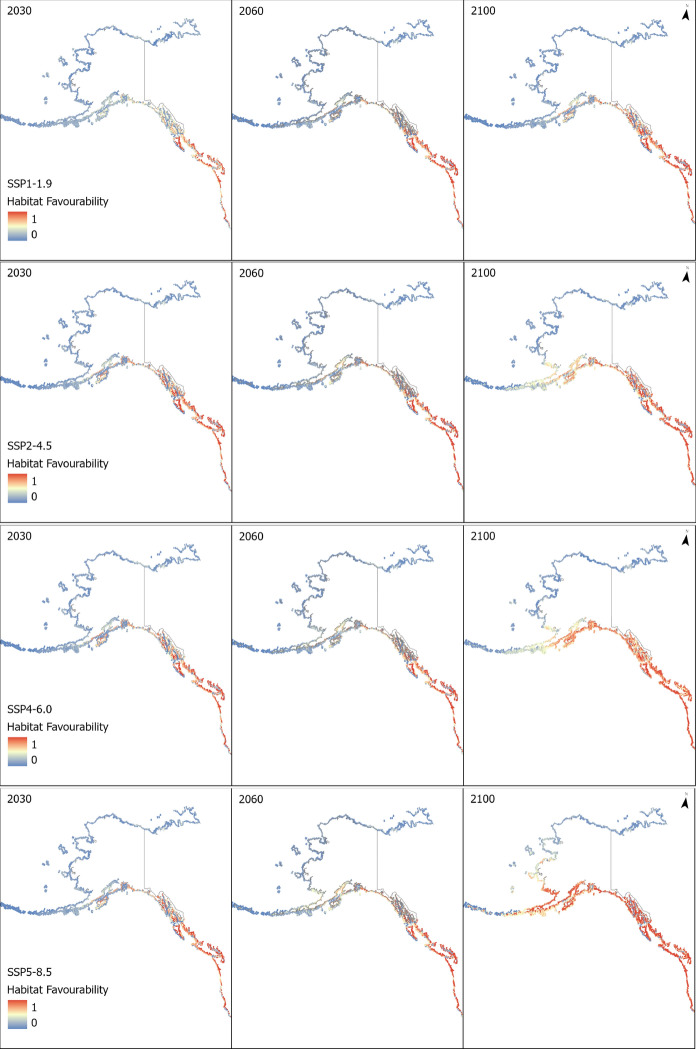
Map of changing habitat favourability for the European Green Crab from 2020 to 2100 under different climate change scenarios

Our primary model had an average Miller’s calibration slope of 1.28, AUC of 0.939, and TSS of 0.886, that all indicate that the model is a good predictor for the distribution and habitat favourability of the European green crab on the Pacific Coast of North America. The environmental predictors of habitat favourability of the European Green Crab are, minimum salinity, mean temperature, and mean productivity. Each explain roughly a third of the model. When comparing the primary model to other models, which used different buffers, numbers of pseudoabsences, and biasing or not biasing said pseudoabsences across all comparisons, only one had a Schoener’s D of under 0.7. That was the comparison of a 50 km buffer and 10,000 unbiased pseudoabsences model with a 50 km buffer with 1000 pseudoabsence model, both for their prediction of green crab distribution under SSP5 in 2100. That had a Schoener’s D of 0.659, which indicates that the models are significantly dissimilar from each other. Under every plausible future climate change scenario, there will be a northwards shift in favourable habitat area of the European green crab on the Pacific Coast of North America (Fig. 2). This increase varies significantly depending both on decade and the climate scenario (Fig. 2). Additionally, our models also suggest that there will be several decades where there will be a decrease in habitat suitability at the southern range limits (Fig. 3).

There will be a slight reduction in the overall favourable region on the Pacific coast of North America in the short term, according to model results (Figs. 2 and 3). This could be due to warming further south in its range making the Californian coast less habitable at a faster rate than the increasing habitat suitability further north. Our model also suggests that, even under present climate conditions, there is a significant swathe of coast north of their current distribution which has potentially favourable habitat for the European green crab (Fig. 3). Under all but the lowest greenhouse gas emissions scenario we can expect the European green crab to find favourable habitat as far north as the Seward Peninsula, and potentially entering into the Arctic Circle. This is a more northerly potential range than predicted by non-model-based expert drawing of potential European green crab ranges (Carlton & Cohen, 2003), and more northerly than predicted ranges from previous SDM-based predictions of the species’ range shift under climate change (Compton et al., 2010; Goldsmit et al., 2018).

## Discussion

This study provides a detailed prediction of the future distribution of the European green crab. Unlike previous SDM-based work (Compton et al., 2010; De Rivera et al., 2011; Goldsmit et al., 2020), we do not draw a hard line between suitable and unsuitable habitat, but use the raw probabilistic habitat suitability values to support our conclusions. Compared to previous work, we suggest that the spread of the European green crab on the Pacific Coast will not be a linear progress, but will be accompanied by declines elsewhere in this part of its invasive range. The northernmost predictions under higher greenhouse gas emission scenarios are further north than those made by previous SDM predictions of European green crab expansion on the Pacific Coast of North America. While these are only predictions of environmental habitat suitability, rather than habitat presence, we do suggest that existing monitoring efforts for European green crab be expanded to these areas to ensure early detection. In addition to greater habitat favourability due to climate change, it is likely that climate change induced greater use of northern shipping lanes will increase the likelihood of European green crab settlement in northern waters (Frederich & Lancaster, 2024). The area around Anchorage is particularly vulnerable, as it already receives significant shipping from ports on the Pacific where the European green crab is commonly found (de Rivera et al., 2011). Arctic waters which might become favourable to the European green crab could also see the species transported there by shipping, especially as the Northwest Passage becomes a more viable commercial shipping route. In British Columbia, the spread of the European green crab has followed major currents (Brasseale et al., 2019)—our model suggests that while currents are important for initial colonisation, they are not determinative of whether a population will be able to establish itself.

While this model only uses data going up to 2020, we can compare the results of our 2030 prediction with range shifts which the European green crab has had since 2020. The results are both encouraging, in that they suggest that our model is accurate, and distressing, in that they suggest that some of our worst-case scenarios may come to pass. The Alaska Department of Fish and Game runs a website which tracks sightings of the Green Crab (Alaska Department of Fish and Game, 2025): as of 2025, the European green crab has been observed as far north as Ketchikan, and appears to be moving tens of kilometres north every year. This is in line with our prediction that the water of the northernmost parts of the Alaskan panhandle will be suitable for the European green crab by the end of the decade.

Early detection is key to preventing further spread of European green crabs (Ens et al., 2022; Frederich & Lancaster, 2024), since only before a population has become established is there any chance at extirpating it locally. Exterminating invasive species is notoriously difficult (Parkes & Panetta, 2009), having only been successfully done a few times, usually at great financial expense, primarily on islands (Glen et al., 2013) or just after an early detection of an invader (Kluever et al., 2023). Exterminating AIS is even more difficult than exterminating terrestrial invasive species (Havel et al., 2015). No attempt to exterminate invasive European green crab has been successful beyond an experimental scale anywhere in the world (Ens et al., 2022; Frederich & Lancaster, 2024). Attempts to reduce European green crab populations from areas with established population, through netting (Beal, 2015), floating cages (Pickering & Quijón, 2011) or fencing off parts of a region to make them European green crab free (Beal, 2015), have failed to have any impact beyond the smallest of spatial and temporal scales. Conversely, some attempts to reduce European green crab population through killing individuals have perversely increased European green crab populations (Grosholz et al., 2021; Tummon Flynn et al., 2024), since removals focused on killing adults who cannibalise larvae and younger crabs. Intense overfishing of this species could lead to functional eradication, with a population low enough to no longer have significant ecological impacts (Tummon Flynn et al., 2024), but this would be a costly programme, dependent either on large governmental budgets or significant numbers of volunteers. These same cost problems also apply to continuous monitoring of the Alaskan coastline.

Should European green crab become established across the Alaskan coastline, this could have potentially devasting impacts on the local economy of the region. Chinook Salmon, Sockeye Salmon *Oncorhynchus nerka* (Walbaum, 1792), and Pacific Herring use eelgrass in different parts of their lifecycle (Shelton et al., 2014; Kennedy et al., 2018; Howard et al., 2019; Sharpe et al., 2019). Alaskan fisheries are amongst the most commercially valuable in the world, with an estimated contribution to the Alaskan economy of 10 billion USD *per annum* (National Marine Fisheries Service, 2024). Salmon fisheries are an estimated 40% of the commercial value of the direct value of the fishery (McKinley Research Group, 2024). Additionally, the shellfish fishery has an estimated value 7% of the direct value of the fishery, if crabs are included in the shellfish category (Ibid.). While it is unclear to what extent European green crab depredation on eelgrass habitats used by Chinook and Sockeye Salmon would have on the populations of these species, any significant population reduction of these species would have deleterious consequences for the Alaskan fishery and economy. Fisheries are also a major industry in British Columbia. As the climate warms and the waters of British Columbia become more suitable for the European green crab, we will likely see them harm that province’s fisheries industry. The British Columbian fisheries industry is worth $548 million Canadian *per annum* and employs 7200 people across the province (Fisheries and Oceans Canada, 2024).

Alaskan and British Columbian Native communities, such as the Tlingit and the Aleut, would likely be amongst the most affected by reductions of salmon fisheries (Reedy-Maschner, 2010; Thornton, 2015; Atlas et al., 2021; Connoy et al., 2024). Members of these native communities engage in salmon and herring fishing both for subsistence and commercially (Reedy-Maschner, 2010; Thornton & Moss, 2021; Connoy et al., 2024). Seafood is crucial for the diet of many Pacific Northwest Native communities (Marushka et al., 2019), and fisheries managers are increasingly encouraged to incorporate indigenous traditional ecological knowledge into management plans (Atlas et al., 2021). The shellfish fishery, while less commercially valuable, is likelier to experience declines due to European green crab. Including crabs, this accounts for an estimated 7% of the commercial value of the Alaskan fishery. Many Native communities harvest shellfish on the Pacific coast of North America (Moss, 1993; Reeder-Myers et al., 2022), though it is less central, both culturally and nutritionally, than salmon.

Establishing a European green crab early warning system (EWS)—systems for hazard monitoring and forecasting in ways that allow advanced actions that reduce risk (UNDRR, 2017)—may be a way to support early detection and rapid response to functionally eradicate the crab. Originally developed for reducing flood, drought, tsunami, and other natural hazard risk (Alcántara-Ayala & Oliver-Smith, 2019), invasive species EWS have been established in many different contexts (FAO, 2007; Magaletti et al., 2018; Reaser et al., 2020; Noar et al., 2021; Srebaliene et al., 2024). EWS is a practical tool, in that it combines robust monitoring with strong communication and action strategies, which is a longstanding weakness in hazard mitigation (Hillbruner & Moloney, 2012). A “last mile” orientation (Kelman & Glantz, 2014) focused on green crab detection and population suppression to the point of functional eradication at the local-level may be particularly helpful for this system, as EWS often fail at the last mile. This could entail citizen science approaches (Marchezini et al., 2018) wherein local fishers and other stakeholders lead the monitoring, detection, and eradication of green crab in their communities with external training, funding, and information sharing support. The use of citizen science to monitor the spread of green crab has been trialled successfully on the local level in several locations globally (Delaney et al., 2008; Grason et al., 2018) but has not been linked with broader systematic early warning and eradication systems. In the United States, such a system could be part of the country’s larger early detection rapid response (EDRR) regime—the widely discussed albeit ambiguously defined and operationalized policy framework for invasive species management (Reaser et al., 2020; Wray et al., 2024).

While SDMs are useful in predicting the habitat favourability of a region for a species, they are not perfect in predicting where a species will be. One of the major drivers of species ranges which are not usually included in SDMs, including in ours, are biotic interactions (Wisz et al., 2013). Biotic interactions, such as competitive exclusion of species with the same niche (Sandro, 2008) or predation (Jensen et al., 2007) are significant drivers of species ranges’ at both a local and a macro scale. In the case of the European green crab, predation is the main biotic interaction which could limit its spread up the Pacific coast of North America. On the Atlantic coast of North America, native Blue Crab *Callinectes sapidus* Rathburn 1896 predates on the European green crab (de Rivera et al., 2005; Rogers et al., 2008; Ens et al., 2022), and regions with high Blue Crab populations tend to have low populations of European green crab (de Rivera et al., 2005; Rogers et al., 2008). On the Pacific coast of North America, the Red Rock Crab *Cancer productus* Randall 1839 predates upon the European green crab (Hunt & Yamada, 2003) and could be a useful biocontrol agent to prevent its spread. High populations of Red Rock Crab could even prevent the establishment of European green crab populations (Hunt & Yamada, 2003; Jensen et al., 2007; Ens et al., 2022), though it is unclear if high populations of Red Rock Crab can extirpate existing populations of European green crab. In addition to these decapod predators of the European green crab, Sea Otters *Enhydra lutris* (L. 1758) and North American River Otters *Lontra canadensis* (Schreber 1777) have been observed to eat European green crab (Kieckhefer et al., 2007; Buzzell, 2021). However, further studies are required to assess whether these mammalian predators can hunt European green crab in sufficient quantities to make them useful biocontrol agents against this invasive species.

SDMs are also only as good as their data. We relied on publicly available biodiversity databases to collect presence points of the European green crab. This is a commonly used data source for biodiversity monitoring and species distribution modelling projects (Heberling et al., 2021), but does come with flaws. These databases have are biased, with species being likelier to be reported if they are charismatic (Dylewski et al., 2025), or readily observed by laypeople (Beck et al., 2014). Much of the data we base our models on comes from relatively well studied or well populated parts of the European green crab’s invasive Pacific Coast range, especially regions around the San Francisco Bay, Victoria Island, and Puget Sound (Fig. 1). It also only covers adult European green crabs, since their planktonic juveniles are not visible to the naked eye and thus unlikely to be recorded by amateur naturalists. Ideally, there would have been a detailed monitoring programme of European green crabs for the past thirty years across every part of its potential range to record both true presences and true absences, at all life stages, and without geographic biases. However, this programme did not exist and is unlikely to ever exist. In the absence of such a programme and data, the use of publicly available biodiversity databases is the standard practice for obtaining the data basis for SDMs, and mitigating biases by integrating data from multiple biodiversity databases (Anderson & Gonzalez, 2011; Dylewski et al., 2025) as we have done in this project.

The scale of this analysis could impact how wildlife managers could use our findings as each pixel was approximately 15.5 by 15.5 km. While this is a relatively small size for the extent of the coastline of a continent, it is a massive area for monitoring and removal of individual European green crabs. The drivers of species presence on a broad scale, such as across a continent and on a fine scale, can vary significantly (Brummer et al., 2013). In European green crabs, it has been shown that on a fine scale, environmental variables such as water temperature and ocean current directions which are the dominant drivers of its distribution on a broad scale, become less significant compared to local topography and the flow of local currents (Cosham et al., 2016). In particular, on the Pacific Coast, the European green crab prefers sheltered coves with plenty of rocks and shellfish (Cosham et al., 2016; Howard et al., 2019). How these variables impact European green crab presence at a local scale also vary depending on whether one is interested in the distribution of adult or juvenile crabs, and on the size of the adult individuals (Cosham et al., 2016). These differences could impact what control methods are used, especially since attempts to prevent the green crab from establishing a population should focus on the harder to detect but more likely to disperse juveniles. Therefore, any localised efforts to monitor European green crab presence, or predict the likelihood that they arrive under distinct climate scenarios, should use finer grained environmental covariates than the ones used in this study. In the absence of finer-scale rasters of relevant environmental variables, wildlife managers could prioritise monitoring efforts on parts of their local coastline which resemble habitats which the European green crab prefers, i.e., protected coves and inlets.

The European green crab is a global problem, not just for the Pacific Coast of North America. This study shows that it is likely to continue to spread north, up to at least the Anchorage region, and very probably up to the Seward Peninsula, if not beyond. This spread could have potentially devasting consequences for local economies and local communities, especially indigenous communities. However, whether the European green crab establishes itself upon the majority of the Pacific Coast of North America is not a certainty; steps can be taken to prevent its spread. Foremost amongst these is a global reduction in greenhouse gas emissions. In lieu of this, constant monitoring for individuals before they establish populations is required to slow the spread of this species, and aid in the early suppression of it before it has significant ecological impacts once it does spread north.

## Supplementary Information

Below is the link to the electronic supplementary material.

Supplementary file1 (CSV 67 KB)

Supplementary file2 (R 119 KB)

## Data Availability

The R code necessary for obtaining the data used in this paper, and its analysis, is available in the supplemental materials of this paper. The resultant species distribution models can be assessed at 10.17605/OSF.IO/FRQSH.
